# Molecular and Functional Analysis of Choline Transporters and Antitumor Effects of Choline Transporter-Like Protein 1 Inhibitors in Human Pancreatic Cancer Cells

**DOI:** 10.3390/ijms21155190

**Published:** 2020-07-22

**Authors:** Kaho Hirai, Saiichiro Watanabe, Nozomi Nishijima, Kaoru Shibata, Akane Hase, Tsuyoshi Yamanaka, Masato Inazu

**Affiliations:** 1 Institute of Medical Science, Tokyo Medical University, 6-1-1 Shinjuku, Shinjuku-ku, Tokyo 160-8402, Japan; rulla.k67@gmail.com (K.H.); saiichiro@gmail.com (S.W.); s22061193@stu.rakuno.ac.jp (N.N.); kaoruinoue08@gmail.com (K.S.); akaneko1123@icloud.com (A.H.); 2 Department of Molecular Preventive Medicine, Tokyo Medical University, 6-1-1 Shinjuku, Shinjuku-ku, Tokyo 160-8402, Japan; yamanaka@rtss.co.jp

**Keywords:** pancreatic cancer, transporter, choline, choline transporter-like protein, ceramide, caspase, apoptosis

## Abstract

Choline, an organic cation, is one of the biofactors that play an important role in the structure and the function of biological membranes, and it is essential for the synthesis of phospholipids. Choline positron emission tomography-computed tomography (PET/CT) provides useful information for the imaging diagnosis of cancers, and increased choline accumulation has been identified in a variety of tumors. However, the molecular mechanisms of choline uptake and choline transporters in pancreatic cancer have not been elucidated. Here, we examined molecular and functional analyses of choline transporters in human pancreatic-cancer cell line MIA PaCa-2 and the elucidation of the action mechanism behind the antitumor effect of novel choline-transporter-like protein 1 (CTL1) inhibitors, Amb4269951 and its derivative Amb4269675. CTL1 and CTL2 mRNAs were highly expressed in MIA PaCa-2 cells, and CTL1 and CTL2 proteins were localized in the plasma membrane and the intracellular compartments, respectively. Choline uptake was characterized by Na^+^-independence, a single-uptake mechanism, and inhibition by choline-uptake inhibitor HC-3, similar to the function of CTL1. These results suggest that the uptake of extracellular choline in MIA PaCa-2 cells is mediated by CTL1. Choline deficiency and HC-3 treatment inhibited cell viability and increased caspase 3/7 activity, suggesting that the inhibition of CTL1 function, which is responsible for choline transport, leads to apoptosis-induced cell death. Both Amb4269951 and Amb4269675 inhibited choline uptake and cell viability and increased caspase-3/7 activity. Ceramide, which is increased by inhibiting choline uptake, also inhibited cell survival and increased caspase-3/7 activity. Lastly, both Amb4269951 and Amb4269675 significantly inhibited tumor growth in a mouse-xenograft model without any adverse effects such as weight loss. CTL1 is a target molecule for the treatment of pancreatic cancer, and its inhibitors Amb4269951 and Amb4269675 are novel lead compounds.

## 1. Introduction

Pancreatic cancer is difficult to differentiate in its early stages and is known to have poor prognosis. Current estimates predict that pancreatic cancer will be the second most lethal tumor by 2030 [1]. Pancreatic ductal adenocarcinoma is the most frequent type of pancreatic cancer, accounting for more than 90% of the cases [2]. For all stages combined, the 5 year relative survival rate is 9% [3]. Patients with pancreatic cancer often have distant metastases and a high recurrence rate after resection, if resection is even possible [4,5]. Combination therapy with fluorouracil, leucovorin, irinotecan, and oxaliplatin (FOLFIRINOX) or gemcitabine is used in patients with metastatic pancreatic cancer [6]. The prognosis of pancreatic cancer has improved since the introduction of gemcitabine and FOLFIRINOX therapies, but results are still unsatisfactory. The development and the clinical application of novel anticancer agents and molecularly targeted drugs against pancreatic cancer are thus required.

Choline is one of the biofactors that play an important role in all cells, essential for the synthesis of phospholipids such as phosphatidylcholine and sphingomyelin. This synthetic pathway is called the Kennedy pathway, which is enhanced in cells with active cell proliferation, such as cancer cells [7]. Choline is also used for the synthesis of acetylcholine and methyl group donor S-adenosylmethionine (SAM). SAM is involved in the methylation of DNA and histones and is involved in epigenetic regulation. Thus, choline is taken up into cells and metabolized into molecules that are important for the organism and are thought to be involved in a variety of physiological functions [7].

Since choline is present in vivo as a water-soluble quaternary ammonium ion, the presence of a transporter is required to cross the cell membrane. Choline transporters are divided into three groups, and each transporter has different properties, including tissue distribution, transport substrate, Na^+^-dependence, and sensitivity to hemicolinium-3 (HC-3), a choline analog that inhibits choline uptake [7]. The first transporter is high-affinity choline transporter 1 (CHT1) that is expressed in cholinergic neurons and is linked to acetylcholine synthesis, which has a high affinity for choline. The second is organic cationic transporters OCT1 and OCT2 that have low affinity for choline, which recognize many cationic substances, and are mainly expressed in the liver and kidney. The last transporter is choline-transporter-like proteins (CTL1-5) that are ubiquitously distributed. Previous studies reported that CTL1 is expressed on the cell membrane and is involved in extracellular choline transport. CTL1-mediated choline transport is thought to be an essential function of phospholipid synthesis, a component of the plasma membrane. Inhibition of these choline-uptake functions was reported to induce apoptotic cell death [8,9,10,11,12]. PET/CT imaging using ^18^F- and ^11^C-choline captures a very high accumulation of choline in tumors [13,14,15]. This choline accumulation in tumors is thought to be related to increased choline uptake due to increased cell-membrane synthesis associated with active cell proliferation, which is reported in many cancers [16,17]. However, the molecular mechanisms of choline uptake in pancreatic cancer are still poorly understood.

Of newly approved drugs between 1981 and 2014, 30% were derived from natural products or their derivatives [18]. Many plant-derived anticancer drugs, such as paclitaxel, vincristine, irinotecan, and etoposide, are currently used in clinical application. Therefore, we searched for compounds that inhibit both CTL1-mediated choline uptake and cell viability in human pancreatic-cancer cells from a library of plant-derived natural organic compounds and discovered isoquinoline derivative Amb4269951 and its derivative Amb4269675 [19]. Recent studies suggested that CTL1 inhibition of Amb4269951 in human glioma cells causes apoptosis-induced cell death by suppressing the expression of apoptosis inhibitory factor survivin via the production of apoptosis-inducing molecule ceramide [20]. Thus, although Amb4269951 has a unique mechanism for cancer treatment, this has never been seen before. Amb4269951 showed the inhibition of cell viability against various cancer cells, most notably, human pancreatic-cancer cell line MIA PaCa-2 [20]. However, the antitumor effects of both Amb4269951 and Amb4269675 in MIA PaCa-2 cells have not been studied.

In this study, we investigated the functional properties of choline uptake and sought to identify the transporter molecules that mediate choline uptake in MIA PaCa-2 cells. We also examined the mechanism of action of the antitumor effects of Amb4269951 and its derivative Amb4269675 and compared the in vivo antitumor effects of both compounds in a mouse-xenograft model.

## 2. Results

### 2.1. Expression of Choline Transporters

**Figure 1:**
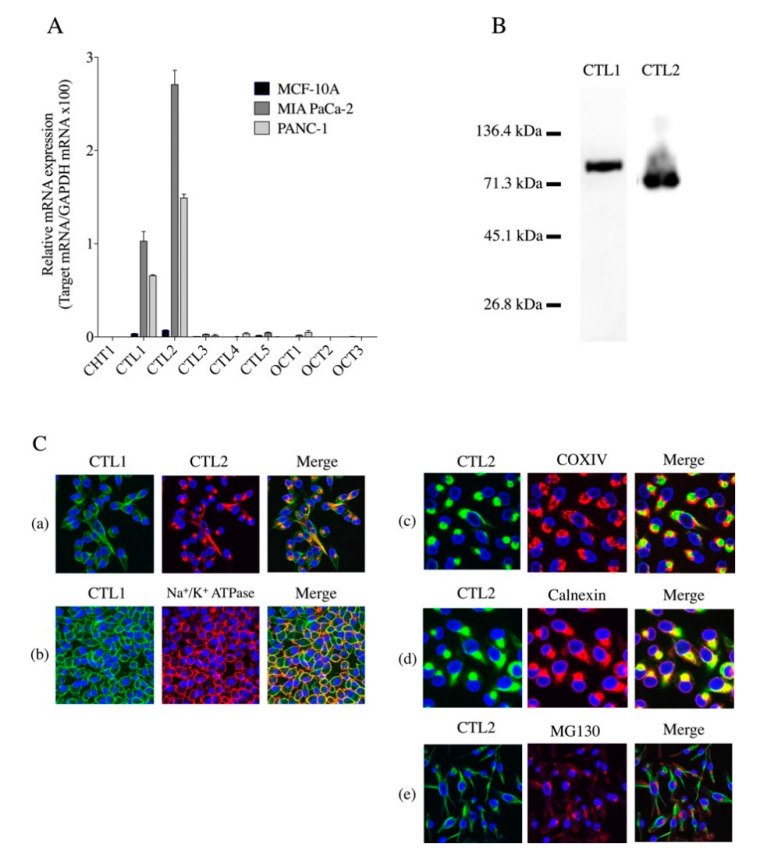
Expression of choline transporters. (**A**) Real-time PCR analysis of mRNA expression of CHT1, CTL1-5, and OCT1,2 in MCF-10A, MIA PaCa-2 and PANC-1 cells (*n* = 3). Relative mRNA expression expressed as ratio of target mRNA to glyceraldehyde-3-phosphate dehydrogenase (GAPDH) mRNA, which is a housekeeping gene. (**B**) Expression of CTL1 and CTL2 proteins in MIA PaCa-2 cells by Western blot analysis. (**C**) Intracellular distribution of CTL1 and CTL2 proteins in MIA PaCa-2 cells. (**Ca**) Subcellular distribution of CTL1 (green) and CTL2 (red) was determined by immunocytochemical staining. DAPI (blue) was used for nuclear staining in all specimens. Merged images labeled Merge, and yellow represents colocalization. (**Cb**) Subcellular distribution of CTL1 protein (green) analyzed using plasma-membrane marker Na^+^/K^+^-ATPase (red). CTL1 protein predominantly present on plasma membrane. Subcellular distribution of CTL2 protein (green) analyzed using mitochondria, endoplasmic reticulum (ER), and Golgi apparatus markers, (**Cc**) COX IV, (**Cd**) calnexin, and (**Ce**) MG130, respectively. CTL2 protein partially localized in mitochondria and ER but not colocalized in the Golgi apparatus.

The mRNA expression of transporters CHT1, CTL1-5, and OCT1,2, which are active transporters of choline in normal human mammary epithelial cell line MCF-10A and human pancreatic-cancer cell lines MIA PaCa-2 and PANC-1, was detected by qPCR (Figure 1A). CTL1 and CTL2 were predominantly highly expressed in MIA PaCa-2 and PANC-1 cells, which were approximately two-fold higher in MIA PaCa-2 cells than in PANC-1 cells. The expression patterns of the choline transporters closely resembled it in both cells. In contrast, CTL1 and CTL2 were only slightly expressed in MCF-10A cells. The expression of other transporters was slight or below the detection limit in these cells. Because mainly CTL1 and CTL2 mRNAs were expressed, we examined their expression at the protein level (Figure 1B). The immunoblotting of extracts of MIA PaCa-2 cell lysates with anti-CTL1 and anti-CTL2 antibodies confirmed the presence of 80 and 70 kDa bands, respectively. The subcellular distribution of CTL1 and CTL2 proteins in MIA PaCa-2 cells was determined using immunocytochemistry (Figure 1C). Immunocytochemical staining with anti-CTL1 and anti-CTL2 antibodies showed that CTL1 was localized on the cell surface, and that CTL2 was subcellularly localized (Figure 1Ca). The immunoreactivity of CTL1 was found to overlap that of plasma-membrane marker Na^+^/K^+^-ATPase (Figure 1Cb). CTL2 immunoreactivity was only found in intracellular organelles, partly overlapping that of mitochondrial marker COX IV (Figure 1Cc) and ER marker calnexin (Figure 1Cd), but not Golgi apparatus marker GM130 (Figure 1Ce).

### 2.2. Effect of CTL1 and CTL2 Expression Levels on Survival of Pancreatic Adenocarcinoma (PAAD) Patients Using a Bioinformatics Analysis

**Figure 2:**
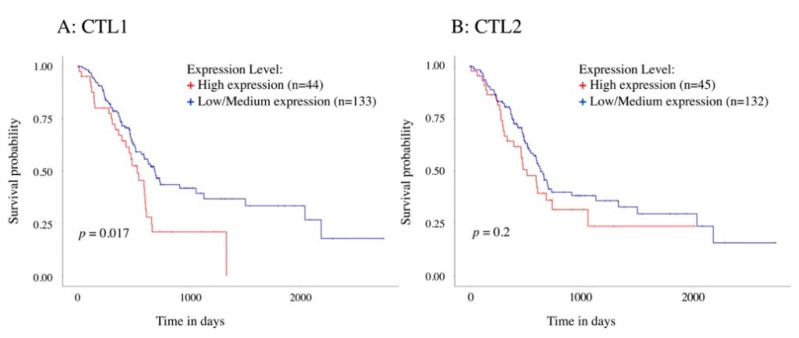
Bioinformatic analysis of association between CTL1 (**A**) and CTL2 (**B**) mRNA expression levels and survival in patients with pancreatic adenocarcinoma (PAAD). Kaplan–Meier plots summarize results from analysis of correlation between mRNA expression level and patient survival. Patients were classified as either high- or low-/medium-expression according to their expression level; x-axis, time of survival (days); y-axis, probability of survival, where 1.0 corresponds to 100%. The *p*-value was calculated using the log-rank test. For CTL1, the difference between the two curves was found to be statistically significant with *p* = 0.017, while for CTL2, the difference between the two curves was not significant (*p* = 0.2).

Kaplan–Meier analysis of overall survival in PAAD patients was performed according to low/medium or high CTL1 and CTL2 mRNA levels; the median of the data was used as the cut-off threshold. CTL1 expression levels and survival were significantly longer in the low-/medium-expression group than those in the high-expression group (Figure 2A). Conversely, we found no significant difference in CTL2 expression levels (Figure 2B). These data suggest that CTL1 has poor prognosis and that a high expression of CTL1 is unfavorable in pancreatic cancer.

Bioinformatics analysis of CTL1 and CTL2 mRNA expression was performed on normal and PAAD patient samples from the Cancer Genome Atlas (TCGA) database (UALCAN website; Figure S1). CTL1 mRNA expression tended to be higher in PAAD patients, whereas CTL2 mRNA expression did not differ from that of normal groups. However, the result was not significant due to the small number in the normal group (*n* = 4).

### 2.3. Properties of [^3^H]Choline Uptake in MIA PaCa-2 and PANC-1 Cells

**Figure 3:**
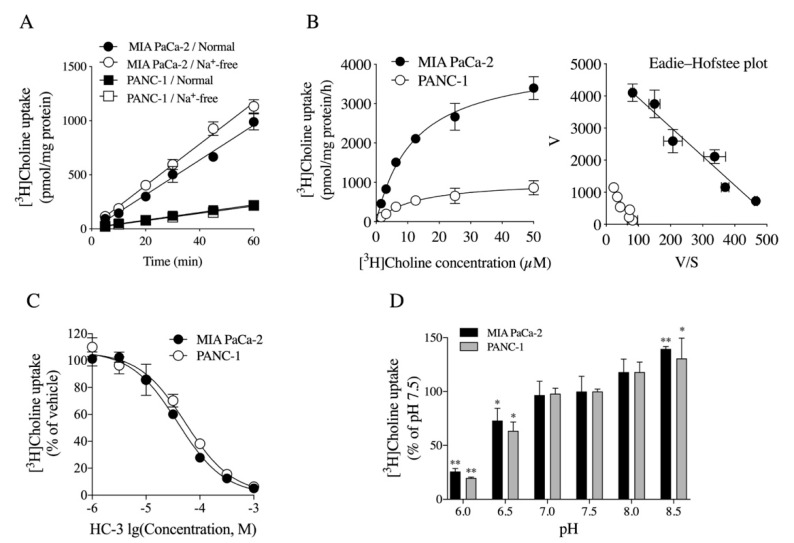
Properties of choline uptake in MIA PaCa-2 and PANC-1 cells. (**A**) Time course and Na^+^ dependence of [^3^H]choline uptake (*n* = 4). Time course of [^3^H]choline uptake fitted using nonlinear-regression analysis. (**B**) Kinetic analysis of [^3^H]choline uptake in both cells (*n* = 4). Cells taken up with [^3^H]choline at concentrations of 1.56–50 µM for 20 min. Kinetic analysis of [^3^H]choline uptake calculated by Michaelis–Menten equation showed *K_m_* of 12.0 ± 1.4 µM and *V_max_* of 4120.0 ± 188.3 pmol/mg protein/h in MIA PaCa-2 cells and *K_m_* of 12.3 ± 3.3 µM and *V_max_* of 1045.0 ± 107.6 pmol/mg protein/h in PANC-1 cells. Eadie–Hofstee plot of [^3^H]choline uptake showing straight line in both cells (MIA PaCa-2 cells; *R*^2^ = 0.9524, *p* = 0.0009, PANC-1 cells; *R*^2^ = 0.8787, *p* = 0.0058). (**C**) Effects of choline-uptake inhibitor HC-3 on [^3^H]choline uptake in MIA PaCa-2 and PANC-1 cells (*n* = 4). Cells incubated with 1 µM to 1 mM HC-3 for 20 min and then 10 µM [^3^H]choline uptake measured for 20 min. IC_50_ value of HC-3 for inhibition of [^3^H]choline uptake in MIA PaCa-2 and PANC-1 cells was 39.1 µM and 54.2 µM, respectively. (**D**) Effect of extracellular pH on [^3^H]choline uptake in MIA PaCa-2 and PANC-1 cells (*n* = 4). Uptake of 10 μM [^3^H]choline measured for 20 min at different conditions from pH 6.0 to pH 8.5; * *p* < 0.05 and ** *p* < 0.01, and one-way ANOVA with Dunnett’s multiple-comparisons test compared to pH 7.5.

CHT1- and CTL1-mediated choline uptake is sodium-dependent and -independent, respectively [7]. Therefore, the time course and the sodium dependence of [^3^H]choline uptake were investigated in MIA PaCa-2 and PANC-1 cells (Figure 3A). [^3^H]choline uptake increased in a time-dependent manner and was not Na^+^-dependent in both cells. The kinetic properties of [^3^H]choline uptake into both cells were also evaluated (Figure 3B). Kinetic analysis of [^3^H]choline uptake, as determined by nonlinear regression analysis, yielded Michaelis–Menten constants (*K_m_*) of 12.0 ± 1.4 µM, and maximal velocities (*V_max_*) of 4120.0 ± 188.3 pmol/mg protein/h in MIA PaCa-2 cells and *K_m_* of 12.3 ± 3.3 µM and *V_max_* of 1045.0 ± 107.6 pmol/mg protein/h in PANC-1 cells (Figure 3B). The Eadie–Hofstee plot shows straight lines in both cells (coefficient of determination (*R*^2^) = 0.9524, *p* = 0.0009 in MIA PaCa-2 cells and *R*^2^ = 0.8787, *p* = 0.0058 in PANC-1 cells). These kinetic data suggested that [^3^H]choline uptake into both cells is mediated by a single transport system with intermediate affinity. Choline-uptake inhibitor HC-3 was reported to completely inhibit the choline-uptake function of CHT1 and CTL1 in the nM and theh µM ranges, respectively [7]. We examined the inhibitory effects of HC-3 on the uptake of [^3^H]choline into MIA PaCa-2 and PANC-1 cells (Figure 3C). The uptake of [^3^H]choline was inhibited by HC-3 in a concentration-dependent manner in MIA PaCa-2 and PANC-1 cells, with IC_50_ values of 39.1 µM and 54.2 µM, respectively. CTL1-mediated choline uptake is pH-dependent and is involved in exchange transport with protons [7,8,9,10,11,12]. Therefore, we investigated the effects of extracellular pH on [^3^H]choline uptake by varying the pH between 6.0 and 8.5 (Figure 3D). [^3^H]Choline uptake was significantly reduced when the extracellular pH was changed to acidic (pH 7.5 to 6.0) and was significantly increased when the extracellular pH was changed to alkaline (pH 7.5 to 8.5) in both cells.

### 2.4. Effects of Choline Deficiency and HC-3 on Cell Viability and Caspase-3/7 Activity in MIA PaCa-2 Cells

**Figure 4:**
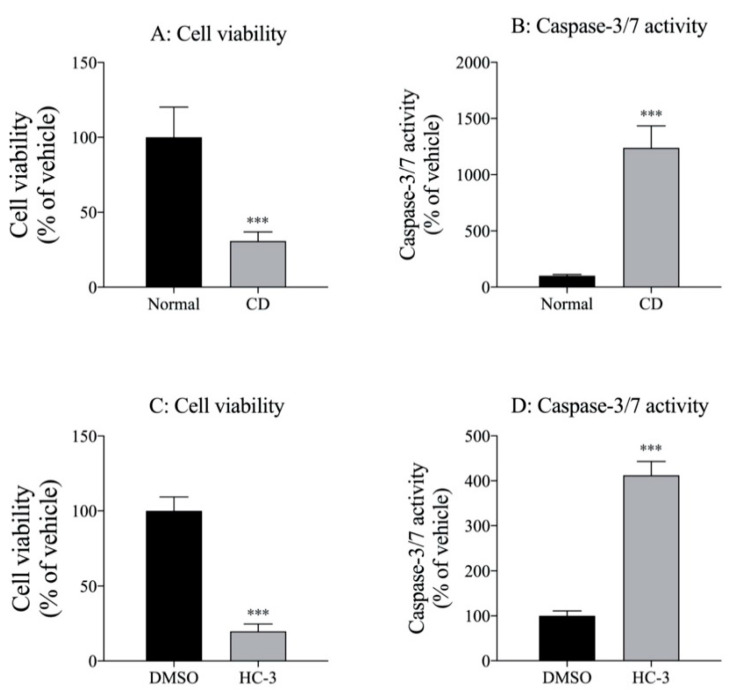
Effect of choline deficiency (CD) on (**A**) cell viability and (**B**) caspase-3/7 activity in MIA PaCa-2 cells. Cells cultured in 30 mM choline chloride (Normal) or without choline chloride (CD) medium for 2 days (*n* = 4). Results calculated as 100% of findings under normal conditions; *** *p* < 0.001 for unpaired *t*-test compared to normal conditions. Effect of HC-3 on (**C**) cell viability and (**D**) caspase-3/7 activity in MIA PaCa-2 cells (*n* = 4). Cells incubated in 1% DMSO (vehicle control) and 1 mM HC-3 for 2 days; then, cell numbers and caspase-3/7 activity were measured. Results calculated as 100% of findings in vehicle control (DMSO); *** *p* < 0.001 for unpaired *t*-test compared to vehicle control (DMSO).

We examined the effects of choline deficiency and the HC-3-induced inhibition of choline transporter functions on cell viability and caspase-3/7 activity in MIA PaCa-2 cells. Cells were incubated in 1 mM HC-3 and Dulbecco’s modified Eagle’s medium (D-MEM) with 30 mM choline chloride (Normal) or without choline chloride (CD) for 48 h. Then, the numbers of cells and caspase-3/7 activity were measured. Choline deficiency (Figure 4A,B) and HC-3 treatment (Figure 4C,D) significantly inhibited cell viability and significantly increased caspase 3/7 activity.

### 2.5. Inhibitory Effect of HC-3, Amb4269951 and Amb4269675 on [^3^H]Choline Uptake in MIA PaCa-2 Cells

**Figure 5:**
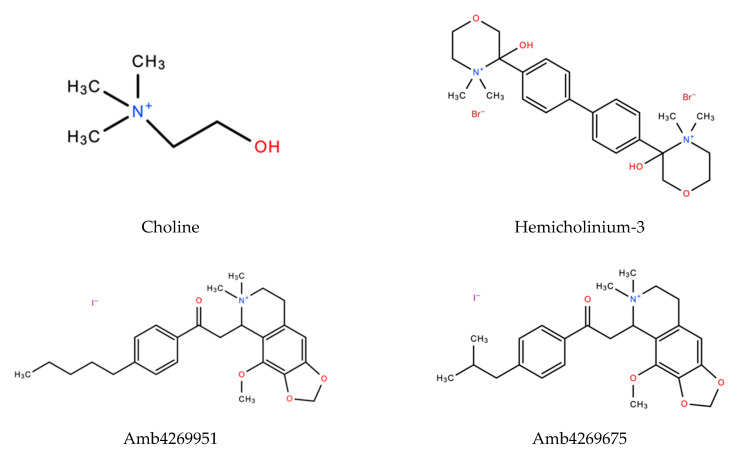
Chemical structure of choline (2-hydroxyethyl(trimethyl)ammonium), hemicholinium-3 (2,2′-(4,4′-biphenylene)bis(2-hydroxy-4,4-dimethylmorpholinium bromide)), Amb4269951 (4-methoxy-6,6-dimethyl-5-(2-oxo-2-(4-pentylphenyl)ethyl)-5,6,7,8-tetrahydro-[1,3]dioxolo[4,5-g]isoquinolin-6-ium iodide), and Amb4269675 (5-(2-(4-isobutylphenyl)-2-oxoethyl)-4-methoxy-6,6-dimethyl-5,6,7,8-tetrahydro-[1,3]dioxolo[4,5-g]isoquinolin-6-ium iodide).

**Figure 6:**
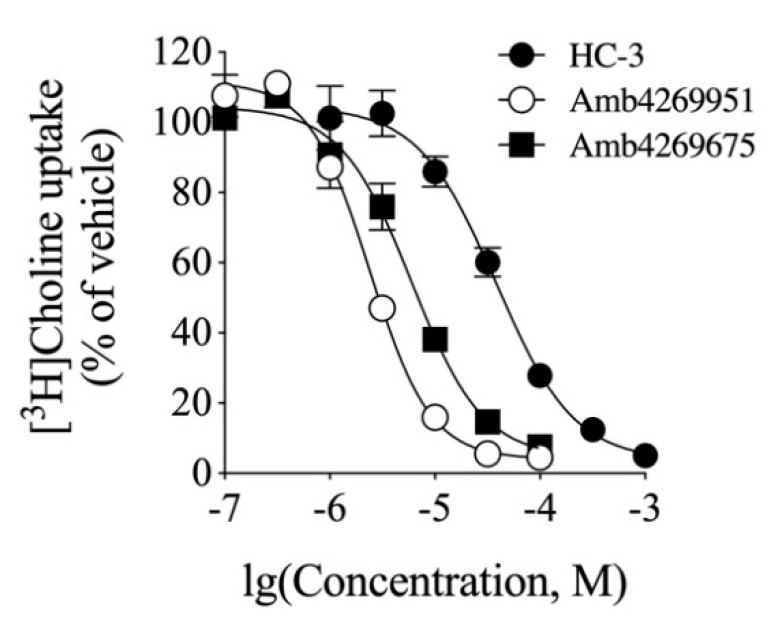
Effect of HC-3, Amb4269951, and Amb4269675 on [^3^H]choline uptake in MIA PaCa-2 cells (*n* = 3). Cells incubated with HC-3, Amb4269951, and Amb4269675 for 20 min and then 10 µM [^3^H]choline uptake measured for 20 min. Results calculated as 100% of findings in vehicle control. IC_50_ values of HC-3, Amb4269951, and Amb4269675 for inhibition of [^3^H]choline uptake were 38.4, 2.4, and 6.0 µM, respectively.

We examined the effects of HC-3, Amb4269951, and Amb4269675 (Figure 5) on [^3^H]choline uptake in MIA PaCa-2 cells (Figure 6). HC-3 is a positive control for choline-uptake inhibitors and was reported to inhibit CTL1-mediated choline uptake in cancer cells [7,8,9,10,11,12]. HC-3, Amb4269951, and Amb4269675 inhibited choline uptake in a concentration-dependent manner, with IC_50_ values of 38.4, 2.4, and 6.0 µM, respectively. Thus, the choline-uptake inhibitory effects of Amb4269951 were 16 and 2.5 times stronger than those of HC-3 and Amb4269675, respectively.

### 2.6. Influence of Amb4269951 and Amb4269675 on Cell Viability and Caspase-3/7 Activity in MIA PaCa-2 Cells

**Figure 7:**
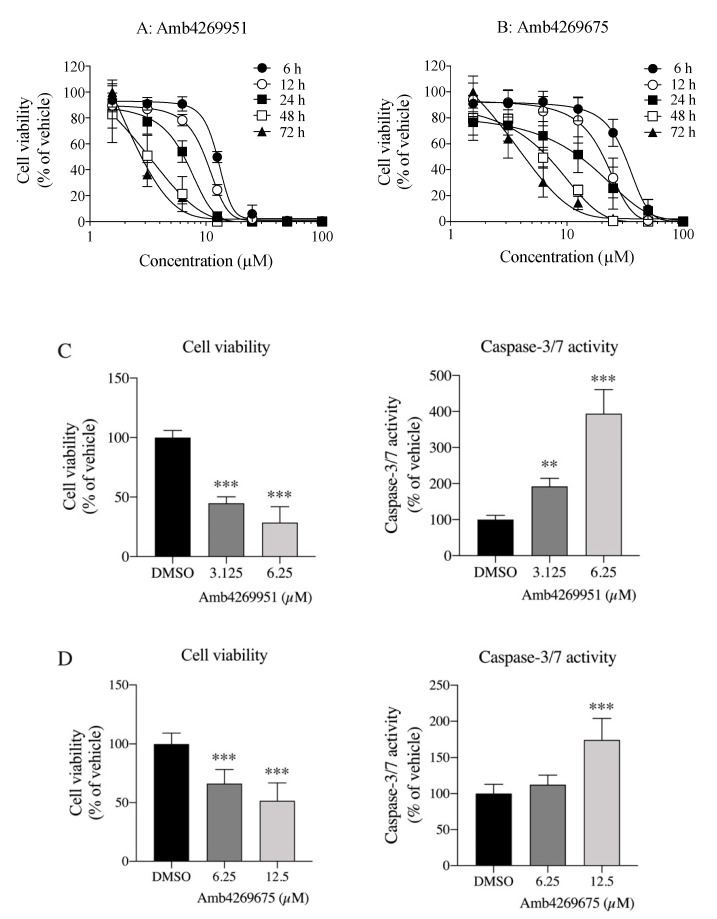
Effects of Amb4269951 and Amb4269675 on cell viability in MIA PaCa-2 cells. Cells incubated with 1.6 to 100 µM (**A**) Amb4269951 and (**B**) Amb4269675 for 6, 12, 24, 48, and 72 h; then, cell numbers were measured (*n* = 3). Results calculated as 100% of findings in vehicle control. Effects of (**C**) Amb4269951 and (**D**) Amb4269675 on cell viability and caspase-3/7 activity in MIA PaCa-2 cells (*n* = 4). Cells incubated with 3.125 and 6.25 µM Amb4269951, and 6.25 and 12.5 µM Amb4269675 for 24 h; then, cell number and caspase-3/7 activity were measured. Results calculated as 100% of findings in vehicle control. ** *p* < 0.01 and *** *p* < 0.001 for one-way ANOVA with Dunnett–s multiple-comparisons test compared to vehicle control (DMSO).

Since Amb4269951 was reported to enhance caspase-3/7 activity and suppress cell viability in glioma cells [20], we examined these effects of Amb4269951 and Amb4269675 in MIA PaCa-2 cells. Both Amb426951 and Amb4269675 inhibited cell viability in a concentration-dependent manner, and their effects were enhanced in a treatment-time-dependent manner (Figure 7A,B). Both Amb4269951 (Figure 7C) and Amb4269675 (Figure 7D) significantly inhibited cell viability and significantly increased caspase-3/7 activity in a concentration-dependent manner.

### 2.7. Effects of Ceramide on Cell Viability and Caspase-3/7 Activity in MIA PaCa-2 Cells

**Figure 8:**
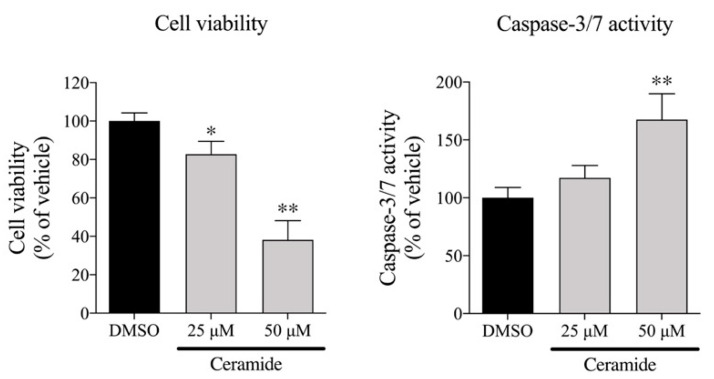
Effects of ceramide on cell viability and caspase-3/7 activity in MIA PaCa-2 cells (*n* = 4). Cells incubated with 25 and 50 µM ceramide for 12 h; then, cell number and caspase-3/7 activity were measured. Results calculated as 100% of findings in vehicle control (DMSO); * *p* < 0.05 and ** *p* < 0.01 for one-way ANOVA with Dunnett’s multiple-comparisons test compared to vehicle control (DMSO).

Inhibition of choline uptake causes a decrease in intracellular choline levels and induces the inhibition of the choline metabolic system, which enhances the degradation of sphingomyelin in cell membranes, resulting in the production of phosphocholine and ceramide. Ceramide is an apoptosis-inducing factor and an increase in ceramide causes apoptosis-induced cell death [21,22]. Therefore, we examined the effects of ceramide on cell viability and caspase-3/7 activity in MIA PaCa-2 cells. Treatment with 25 and 50 µM ceramide for 12 h significantly decreased cell viability and significantly increased caspase 3/7 activity in a concentration-dependent manner (Figure 8).

### 2.8. Effects of Amb4269951 and Its Derivative Amb4269675 in MIA PaCa-2 Cell Mouse-Xenograft Model

**Figure 9:**
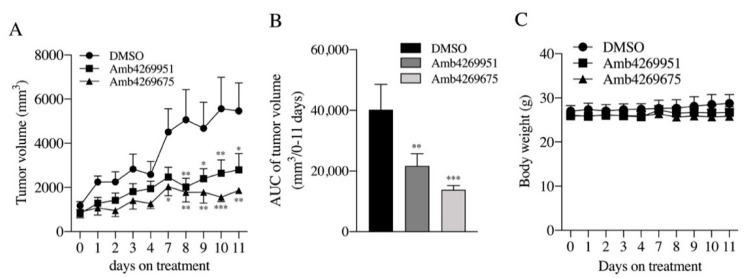
Effects of Amb4269951 and Amb4269675 on MIA PaCa-2 xenograft tumor progression and body weight. (**A**) Amb4269951 (10 mg/kg, *n* = 5 mice), Amb4269675 (10 mg/kg, *n* = 5 mice), and 100% DMSO as solvent control (*n* = 5 mice) were injected intraperitoneally on days 0–4 and 7–11. Tumor volume measured prior to each administration of test compounds; * *p* < 0.05, ** *p* < 0.01, and *** *p* < 0.001 for a two-way ANOVA with Sidak’s multiple-comparisons test compared to vehicle control (DMSO). (**B**) Area under curve (AUC) of tumor volume in each group over 0–11 days (*n* = 5 mice); ** *p* < 0.01 and *** *p* < 0.001 for one-way ANOVA with Dunnett’s multiple-comparisons test compared to vehicle control (DMSO). (**C**) The body weight of each group was measured at each drug administration (*n* = 5 mice). There were no significant differences between groups throughout all treatment periods.

To evaluate the in vivo antitumor effects of both Amb4269951 and Amb4269675, mice in an MIA PaCa-2 cell xenograft model were intraperitoneally injected with 10 mg/kg Amb4269951 and 10 mg/kg Amb4269675. Continuous administration of Amb4269951 and Amb4269675 significantly inhibited tumor growth from days 7 to 11 (Figure 9A), and the area under the curve (AUC) of the tumor volume was significantly inhibited from days 0 to 11 (Figure 9B). The antitumor effect of Amb4269675 was stronger than that of Amb4269951. No weight loss was observed for either compounds compared to the DMSO control group (Figure 9C).

## 3. Discussion

Studies of the mechanism of choline uptake in cancer cells showed that increased choline uptake causes an increase in the amount of intracellular phosphocholine (a phosphorylated compound of choline). This phosphocholine is used for the synthesis of phosphatidylcholine and is an essential molecule for the synthesis of cell membranes. Elevated levels of phosphocholine and choline in cancer cells characterize many cancer cells [16,23,24]. The biosynthesis of phosphatidylcholine was reported to be an essential process for the promotive signaling events of cell division [25]. In pancreatic cancer, tumor tissue was reported to have higher choline content than normal tissue when studied using ^1^H magnetic-resonance spectroscopic imaging (MRSI) and MRS, suggesting abnormalities in the choline metabolic system [26,27]. However, the function of choline transport and the identity of choline transporter molecules in pancreatic cancer have not been fully elucidated.

In this study, we first analyzed the molecular and the functional characteristics of choline transporters in the human pancreatic-cancer cell lines MIA PaCa-2 and PANC-1. CTL1 and CTL2 mRNAs were predominantly expressed in both cells. CTL1 and CTL1 mRNA expression levels in these pancreatic cancer cells were higher than those in the normal human mammary epithelial cell line MCF-10A. In addition, CTL1 mRNA expression tended to be higher in PAAD patients by bioinformatics analysis. These findings suggest that high CTL1 expression may be a feature of pancreatic cancer. CTL1 was localized to the plasma membrane, whereas CTL2 was localized to the endoplasmic reticulum (ER) and mitochondria. These results suggested that CTL1 is responsible for extracellular choline transport, whereas CTL2 is responsible for choline transport in the ER and mitochondria. The primary fate of choline is that it is either phosphorylated and used to make phospholipids, the main component of cell membranes, or it is oxidized and used as a SAM, a donor of methyl groups. The former phosphorylation pathway is predominantly conducted in the ER, whereas the latter occurs in the mitochondria [28]. Thus, CTL2 may be deeply involved in different biological functions but is mainly involved in the supply of choline to the ER and mitochondria, which are intracellular organelles.

Functional characterization of choline uptake in MIA PaCa-2 and PANC-1 cells revealed a single, sodium-independent uptake mechanism. Choline uptake was completely inhibited by HC-3, a choline-uptake inhibitor with an IC_50_ value of 39.1 µM. These choline-uptake functions are similar to those of CTL1 [7] and are localized to the plasma membrane, leading us to conclude that extracellular choline uptake is mediated by CTL1.

Studies showed that the inhibition of choline uptake in cancer cells, such as those of lung, tongue, and esophageal cancers, leads to apoptosis-induced cell death [8,9,12]. Rat adrenal pheochromocytoma cell line PC12 induces apoptosis when cultured in a choline-free medium, resulting in decreased phosphatidylcholine and sphingomyelin concentrations [29]. A decrease in intracellular choline concentration causes apoptosis. Therefore, we investigated the cell viability and the apoptosis induced by choline deficiency and choline-uptake inhibitor HC-3 in MIA PaCa-2 cells. We found that choline deficiency and HC-3 treatment inhibited cell viability and increased caspase 3/7 activity in MIA PaCa-2 cells. These findings suggested that the inhibition of CTL1 function, which is responsible for choline transport in MIA PaCa-2 cells, leads to apoptosis-induced cell death. Using the TCGA database, Kaplan–Meier analysis showed that CTL1 is associated with a poor prognosis, with high expression being unfavorable in pancreatic cancer. These results suggested that CTL1-mediated choline uptake is enhanced in various cancer cells, promotes the synthesis of phospholipids of the cell membrane, and is used for cell proliferation. Therefore, CTL1 appears to be a novel target molecule for cancer therapy.

Recently, we discovered isoquinoline derivative Amb4269951 and its derivative, Amb4269675, from a library of plant-derived natural organic compounds during the search for compounds that inhibit both CTL1-mediated choline uptake and cell viability in MIA PaCa-2 cells [19]. We reported that Amb4269951 causes apoptosis-induced cell death in human glioma cells by suppressing the expression of apoptosis inhibitor survivin through the production of ceramide caused by CTL1-mediated inhibition of choline uptake [20]. However, neither Amb4269951 nor Amb4269675 have been studied in pancreatic-cancer cells. We investigated the mechanism of the antitumor effect of Amb4269951 and Amb4269675 in MIA PaCa-2 cells. The inhibitory effects of Amb4269951 and Amb4269675 on choline uptake were compared with those of choline-uptake inhibitor HC-3. The inhibitions of choline uptake by both Amb4269951 and Amb4269675 were stronger than that of HC-3, and that of Amb4269951 was twice as strong as that of Amb4269675. We previously reported that choline is a cationic compound under physiological conditions and that CTL1-mediated choline uptake is inhibited by organic cationic compounds [7,8,9]. Since choline has the trimethyl moiety of the cation, and HC-3, Amb4269951, and Amb4269675 have the dimethyl moiety of the cation, it is possible that these cationic sites serve as the recognition sites for CTL1. It is necessary to examine the structure–activity relationship of the cation site with structural modifications to reveal the recognition site of CTL1.

Acidic extracellular pH is a major feature of tumor tissue [30]. Acidic extracellular pH is maintained in cancer cells through the increased expression or activity of plasma membrane ion transporters and intracellular pH regulators, including the Na^+^/H^+^ exchanger 1 (NHE1), carbonic anhydrases, monocarboxylate transporter 1 and 4, and Na^+^-driven HCO_3_^−^ exchangers [31]. These properties are associated with tumor growth, invasion, metastasis, aggressiveness, and resistance to treatment [32]. We found that choline uptake was significantly reduced by acidification of extracellular pH in MIA PaCa-2 and PANC-1 cells. These data suggest that choline may be transported by a choline/H^+^ antiport system using a directed H^+^ gradient as a driving force. Thus, CTL1-mediated choline uptake may contribute to the acidification of extracellular pH. Previous studies have reported that CTL1 is functionally expressed in neuroblastoma cells and is involved in H^+^ gradient-dependent choline uptake as a driving force, and that this transport functions in cooperation with NHE1 [33]. The antitumor effects of both Amb4269951 and Amb4269675 may be due in part to the inhibition of extracellular pH acidification by CTL1 inhibition.

Prostate-cancer drugs flutamide and bicalutamide inhibit both cell viability and choline uptake and increase caspase-3/7 activity in prostate-cancer cells. In addition, these drugs decrease CTL1 function and its expression [10]. Other studies showed that the CTL1-mediated choline uptake in glioma cells is inhibited by existing anticancer drugs vincristine, cisplatin, etoposide, and temozolomide [8]. Thus, the CTL1-mediated choline transport system in cancer cells may be a novel therapeutic target for cancer therapy. In this study, we found that both Amb4269951 and Amb4269675 can inhibit cell viability and enhance caspase-3/7 activity in MIA PaCa-2 cells. The inhibitory effect of Amb4269951 and Amb4269675 on cell viability was enhanced in a treatment-time-dependent manner. Time-dependent suppression of cell viability by Amb4269951 was also observed in U251MG glioma cells [20]. These effects of both compounds may be due to the inhibition of choline uptake, which inhibits choline metabolic systems, including the Kennedy pathway, and thus suppresses phospholipid synthesis in the cell membrane. Another possibility is that the cell death caused by Amb4269951 and Amb4269675 may release lactic acid, which acidifies the extracellular pH and further enhances the inhibition of choline uptake. For these compounds to effectively work, they must be continuously administered over a long period of time.

It is well known that acetylcholine can stimulate cell growth through either nicotinic or muscarinic cholinergic pathways [34,35]. The presence of non-neural acetylcholine production has been reported in lung cancer cells, and released acetylcholine stimulates cell proliferation via the autocrine and paracrine systems [12,34]. Therefore, it is possible that cell proliferation is regulated by the non-neuronal acetylcholine system in MIA PaCa-2 cells. However, MIA PaCa-2 cells did not express choline acetyltransferase (ChAT) mRNA (Figure S2), suggesting the absence of a non-neural acetylcholine system. Therefore, the non-neuronal ACh system may not be involved in the antitumor effects of Amb4269951 and Amb4269675.

Inhibition of choline uptake results in a decrease in the phosphocholine and phosphatidylcholine of the plasma-membrane components due to a decrease in intracellular choline. Cancer cells hydrolyze sphingomyelin as a compensatory response to prevent phosphocholine and phosphatidylcholine reduction and promote the production of phosphatidylcholine and apoptosis-inducing factor ceramide. As a result, intracellularly produced ceramides activate caspase-mediated apoptotic signals and induce cell death [7,36,37]. Choline deprivation was also reported to decrease phosphocholine and phosphatidylcholine, increase ceramide production, and induce apoptosis [29]. In recent years, research has treated cancer by manipulating the production and the metabolism of ceramide [36,37,38]. Ceramide treatment of cells inhibited cell viability and increased caspase-3/7 activity in both MIA PaCa-2 and U251MG glioma cells [20]. These results suggested that the antitumor activity of both Amb4269951 and Amb4269675 may exert apoptotic-induced cell death by ceramide-mediated enhanced caspase-3/7 activity. In the future, LC-MS/MS analysis is required to verify that treatment with these compounds promotes ceramide production in MIA PaCa-2 cells.

The in vivo antitumor effects of Amb4269951 and Amb4269675 were compared using a mouse-xenograft model. Both Amb4269951 and Amb4269675 inhibited tumor growth, and no weight loss was observed. Classic antitumor drugs such as cisplatin were reported to cause severe weight loss [39], suggesting that these compounds may not be of concern for severe weight loss. Amb4269951 also showed antitumor effects without weight loss in U251MG glioma cells [20]. Comparing the antitumor effects of both compounds, the tumor-growth inhibition of Amb4269675 was stronger than that of Amb4269951. Despite the inhibition of both choline uptake and cell viability in vitro being twice stronger with Amb4269951 than with Amb4269675, the in vivo effect was the opposite. Increasing the lipophilicity of a compound often improves its pharmacological activity against the target. On the other hand, compounds that have high lipophilicity are prone to pharmacokinetic problems. What is important is the balance between activity and lipophilicity, and lipophilic efficiency (LipE = pIC_50_-logP) is a good reference for this [40]. Therefore, we calculated the LipE of Amb4269951 and Amb4269675 to be 3.5 and 3.7, respectively, and found that Amb4269675 is a more effective compound in vivo. Differences in the in vivo antitumor effects of both compounds may contribute to their pharmacokinetic profiles. Analysis of the pathology of malignant tumors and the development of new therapies requires an appropriate animal-model analysis system. Recently, generating patient-derived tumor xenografts (PDXs) has become easier. These PDXs retain the characteristics of patient-derived tumors, thus they are expected to be used for the pathological analysis of tumors, stem-cell and tumor-marker analysis, and drug development [41]. In the future, PDX models should be used to evaluate the antitumor effects of Amb4269951 and Amb4269675.

## 4. Materials and Methods

### 4.1. Cell Culture

Human pancreatic-cancer cell lines MIA PaCa-2 (RCB2094) and PANC-1 (RCB2095) were purchased by RIKEN BRC through the National Bio-Resource Project of the MEXT/AMED, Ibaraki, Japan. MIA PaCa-2 and PANC-1 cells were cultured in Dulbecco’s modified eagle medium (D-MEM; Life Technologies Corporation, Carlsbad, CA, USA) supplemented with 10% fetal bovine serum (Biowest SAS, Nuaillé, France), 100 units/mL penicillin, and 100 µg/mL streptomycin. Cells were maintained in a humidified incubator with 95% air and 5% CO_2_ atmosphere at 37 °C. Normal human mammary epithelial cell line MCF-10A (ATCC^®^ CRL-10317^TM^) was purchased from American Type Culture Collection (ATCC^®^, Manassas, VA, USA). MCF-10A cells were cultured in a 1:1 mixture of Dulbecco’s modified Eagle’s medium and F12 medium (DMEM-F12; Life Technologies Corporation, Carlsbad, CA, USA) supplemented with 10% fetal bovine serum, 0.5 µg/mL hydrocortisone (Sigma-Aldrich, St. Louis, MO, USA), 10 µg/mL insulin (Invitrogen, Carlsbad, CA, USA), and 20 ng/mL recombinant human fibroblast growth factor (Invitrogen, Carlsbad, CA, USA).

### 4.2. Isoquinoline Derivatives Amb4269951 and Amb4269675

Amb4269951 (4-methoxy-6,6-dimethyl-5-(2-oxo-2-(4-pentylphenyl)ethyl)-5,6,7,8-tetrahydro-[1,3]dioxolo[4,5-g]isoquinolin-6-ium iodide) and Amb4269675 (5-(2-(4-isobutylphenyl)-2-oxoethyl)-4-methoxy-6,6-dimethyl-5,6,7,8-tetrahydro-[1,3]dioxolo[4,5-g]isoquinolin-6-ium iodide) were obtained from Greenpharma SAS (Orléans, France). Both Amb4269951 and Amb4269675 were dissolved in 100% DMSO, and the final concentration was used as 1%.

### 4.3. mRNA Quantification by Reverse Transcription Polymerase Chain Reaction (RT–PCR)

RT-PCR analysis was performed as previously described [8,9,10]. MCF10A, MIA PaCa-2, and PANC-1 cells were homogenized with QIAshredder (Qiagen, Venlo, The Netherlands), and total RNA was purified using the RNeasy^®^ Mini Kit (Qiagen, Venlo, The Netherlands). TaqMan probes for the target mRNAs were designed using TaqMan^®^ Gene Expression Assays (Applied Biosystems, Foster City, CA, USA). Assay IDs of the TaqMan probes used were: CHT1, Hs00222361_m1; CTL1, Hs00223114_m1; CTL2, Hs01105936_m1; CTL3, Hs00537043_m1; CTL4, Hs00228901_m1; CTL5, Hs01120485_m1; OCT1, Hs00427552_m1; OCT2, Hs01010726_m1; glyceraldehyde-3-phosphate dehydrogenase (GAPDH), Hs02786624_m1 [8,9,10]. Real-time PCR was measured using the TaqMan^®^ RNA-to-CT^TM^ 1-Step Kit (Applied Biosystems, Foster City, CA, USA), and data analysis was performed using a LightCycler^®^ 96 system (Roche Diagnostics, Mannheim, Germany). The mRNA expression level relative to glyceraldehyde-3-phosphate dehydrogenase (GAPDH), the housekeeping gene, for each target gene was calculated according to the comparative threshold-cycle (C_t_) method [8,9,10].

### 4.4. Western Blot Analysis

Western blot analysis was performed as previously described [8,9,10,11]. MIA PaCa-2 cells in a lysis buffer containing 2 mM phenylmethylsulfonyl fluoride, 1 mM ethylenediaminetetraacetic acid, and a Halt^TM^ protease inhibitor cocktail (Thermo Fisher Scientific Pierce Biotechnology, Rockford, IL, USA) were homogenized on ice by ultrasonic disruption, then centrifugated at 14,000× *g* for 15 min at 4 °C. The supernatant was mixed in an equal volume of Tris-SDS β-ME sample solution for 10 min at 100 °C and then electrophoresed on a 10% Mini-PROTEAN^®^ TGX^TM^ Gel (BioRad Laboratories, Inc., Hercules, CA, USA). The separated proteins were transferred onto polyvinylidene fluoride membrane using a Trans-Blot^®^ Turbo^TM^ Transfer System (BioRad Laboratories, Inc.), and membranes were blocked with iBind^TM^ Flex Solution (Thermo Fisher Scientific Inc., Waltham, MA, USA) [8,9,10,11]. Membranes were then incubated with 4 µg/mL anti-CTL1 (ab110767, Abcam plc, Cambridge, U.K.) and anti-CTL2 (clone 3D11, Abnova Corporation, Taipei, Taiwan) antibodies in an iBind^TM^ Flex Solution for 4 h at room temperature. Membranes were then washed three times every 5 min with the iBind^TM^ Flex Solution and incubated with 1 µg/mL horseradish peroxidase (HRP)-conjugated secondary antibody (HRP-conjugated anti-rabbit IgG; 214-1516, HRP-conjugated anti-mouse IgG; 214-1806, Kirkegaard and Perry Laboratories Inc., Gaithersburg, MD, USA) for 1 h at room temperature. Membranes were then washed three times every 5 min with the iBind^TM^ Flex Solution, and bands were detected using an ECL^TM^ Prime Western Blotting Detection System (GE Healthcare Life Sciences, Buckinghamshire, UK). Chemiluminescent images were acquired using a ChemiDoc XRS^+^ System (Bio-Rad Laboratories, Hercules, CA, USA).

### 4.5. Immunocytochemistry

Subcellular localization of CTL1 and CTL2 proteins using immunocytochemistry was performed as previously described [8,9,42]. MIA PaCa-2 cells cultured on a 35 mm noncoated Glass Base dish (IWAKI, Tokyo, Japan) were fixed with 100% methanol at −20 °C for 20 min. Fixed cells were incubated with the iBind^TM^ Flex Solution for 1 h at room temperature. CTL1 localization to the plasma membrane was studied using the anti-Na^+^/K^+^-ATPase antibody (Abcam plc, Cambridge, UK). Fixed cells were incubated with 2 µg/mL anti-CTL1 (ab110767, Abcam plc, Cambridge, UK) and 2 µg/mL anti-Na^+^/K^+^-ATPase antibodies for 4 h at room temperature. Organelle marker antibodies were used to confirm the colocalization of CTL2 with mitochondria, endoplasmic reticulum (ER), and Golgi apparatus. The primary antibodies listed below were incubated for 4 h at room temperature: 2 µg/mL anti-CTL2 antibody (clone 3D11, Abnova Corporation, Taipei, Taiwan), 1 µg/mL anti-COX IV antibody (ab202554, Abcam plc, Cambridge, UK), 2 µg/mL anticalnexin antibody (PM060MS, Medical Biological Laboratories Co., Ltd., Nagoya, Japan), and 1:250 anti-MG130 antibody (PM061, Medical Biological Laboratories Co., Ltd., Nagoya, Japan). Excess antibodies were washed three times every 5 min with the iBind^TM^ Flex Solution; they were then incubated for 1 h at room temperature with the following secondary antibodies: 1 µg/mL Alexa Fluor 488 goat antirabbit IgG (A-11008, Molecular Probes Inc, Eugene, OR, USA), 1 µg/mL Alexa Fluor 488 goat antimouse IgG (A-11001, Molecular Probes Inc., Eugene, OR, USA), 1 µg/ mL Alexa Fluor 568 goat antimouse IgG (A-11004, Molecular Probes Inc., Eugene, OR, USA), and 1 µg/mL Alexa Fluor 568 goat antirabbit IgG (A-11036, Molecular Probes Inc., Eugene, OR, USA). Excess antibodies were washed three times every 5 min with the iBind^TM^ Flex Solution and mounted using a VECTASHIELD mounting medium with DAPI (Vector Laboratories, Inc., Burlingame, CA, USA). Fluorescence images were acquired using a fully automated confocal laser-scanning microscope (FLUOVIEW FV10i-DOC, Olympus, Tokyo, Japan).

### 4.6. Bioinformatics Analysis

The association between CTL1 and CTL2 mRNA expression levels and survival in patients with pancreatic adenocarcinoma (PAAD) was analyzed via specific queries on the UALCAN, a bioinformatics web portal, on the basis of the Cancer Genome Atlas (TCGA) and Clinical Proteomic Tumor Analysis Consortium (CPTAC) datasets (http://ualcan.path.uab.edu) [43]. Analysis of the correlation between CTL1 and CTL2 mRNA expression levels and patient survival was analyzed using Kaplan–Meier plots. Patients were classified into either high- or low-/medium-expression groups according to their expression level.

### 4.7. [^3^H]Choline Uptake in MIA PaCa-2 Cells

Choline-uptake experiments were performed as previously described [8,9,10,11,12]. MIA PaCa-2 and PANC-1 cells were cultured in 24-well plates at 1 × 10^5^ cells/well. [^3^H]choline-uptake experiments were performed 24 h after cell culture. Cells were washed twice with an uptake buffer (125 mM NaCl, 1.2 mM CaCl_2_, 4.8 mM KCl, 1.2 mM MgSO_4_, 1.2 mM KH_2_PO_4_, 5.6 mM glucose, and 25 mM HEPES, pH 7.4). Choline uptake was initiated by adding [^3^H]choline to cells containing the uptake buffer. The [^3^H]choline uptake reaction was stopped by washing with a precooled uptake buffer. Cells were lysed in 0.1% Triton X-100/0.1 N NaOH solution, and radioactivity was measured using a liquid-scintillation counter (Tri-Carb1 2100 TR, Packard Instrument Company, Meriden, CT, USA). Na^+^-free buffer was prepared using equimolar N-methyl-D-glucamine chloride (NMDG-Cl) instead of NaCl. The different pH uptake buffers were prepared by mixing solutions adjusted to pH 6.0 with 25 mM 2-(*N*-morpholino) ethanesulfonic acid, and to pH 8.5 with 25 mM Tris. Both uptake buffers contained 125 mM NaCl, 1.2 mM CaCl_2_, 4.8 mM KCl, 1.2 mM MgSO_4_, 1.2 mM KH_2_PO_4_, and 5.6 mM glucose [8,9,10,11,12].

### 4.8. Cell-Viability Assay

The cell-viability assay was performed as previously described [8,9,10,20]. MIA PaCa-2 cells were cultured in 96-well plates with 1000 cells/well. Amb4269951, Amb4269675, and ceramide (Cayman Chemical, Ann Arbor, MI, USA) were added 24 h after cell culture. The final concentration of DMSO used as solvent was set at 1%. Cell numbers were measured using a CellTiter-Glo^®^ Luminescent Cell Viability Assay (Promega, Madison, WI, USA). Chemiluminescence measurements were performed using a FilterMax^TM^ F5 Multi-Mode Microplate Reader (Molecular Devices, LLC, Sunnyvale, CA, USA).

### 4.9. Measurements of Caspase-3/7 Activity

Caspase 3/7 activity was performed as previously described [8,9,10,20]. MIA PaCa-2 cells were cultured in 96-well plates with 1000 cells/well. Amb4269951, Amb4269675, and ceramide were added 24 h after cell culture. The final concentration of DMSO used as solvent was set at 1%. Caspase-3/7 activity and cell numbers were simultaneously measured using Caspase-Glo^®^ 3/7 Assay (Promega, Madison, WI, USA) and CellTiter-Glo^®^ Luminescent Cell Viability Assay (Promega, Madison, WI, USA), respectively. Caspase 3/7 activity was calculated as activity per viable cell [20]. Chemiluminescence measurements were performed using a FilterMax^TM^ F5 Multi-Mode Microplate Reader (Molecular Devices, LLC., San Jose, CA, USA).

### 4.10. Mouse-Xenograft Model

In vivo evaluation of the mouse-cancer-cell xenograft model was performed as previously described [20]. All animal experiments were approved by the Animal Research Committee of Tokyo Medical University (approval number H31-0044). Five mice per cage were housed under pathogen-free conditions. A suspension of MIA PaCa-2 cells (1 × 10^7^ cell/0.2 mL/mice) was injected subcutaneously into the dorsal region of 6-week-old male BALB/cAJcl-nu/nu mice (CLEA Japan, Inc., Shizuoka, Japan) to produce solid tumors. The grown solid tumors were cut into 3 mm square blocks under sterile conditions and implanted into the dorsal region of 6-week-old male BALB/cAJcl-nu/nu mice. Five mice per group were used. The body weight, length (L, mm), and width (W, mm) diameters of the tumor were measured prior to each administration of the test compounds; tumor volume was calculated on the basis of the following formula: volume (mm^3^) = L × W^2^ × 1/2 [17]. Intraperitoneal administration of the test compounds was initiated after the tumor volume reached approximately 500–1000 mm^3^. Amb4269951, Amb4269675, and 100% DMSO as solvent control were injected intraperitoneally (0.1 mL/mice) on days 0–4 and 7–11.

### 4.11. Data Analysis

Data were expressed as the means ± SD. Statistical analyses were performed with an unpaired *t*-test for two groups, one-way ANOVA with Dunnett’s multiple-comparisons test, and two-way ANOVA with Sidak’s multiple-comparisons test for data from multiple groups using statistical analysis software InStat 3 and Prism 8 (GraphPad Software, Inc., San Diego, CA, USA). When the *p*-value was less than 0.05, results were considered statistically significant. The kinetic parameters calculated by the Michaelis–Menten equation and the Eadie–Hofstee plot were performed using analysis software Prism 8.

## 5. Conclusions

These findings revealed that CTL1 is functionally expressed in MIA PaCa-2 cells and is involved in extracellular choline uptake. Results further revealed that CTL1 is also a prognostic factor in pancreatic cancer. Isoquinoline derivatives Amb4269951 and Amb4269675, discovered through exploratory studies using a library of plant-derived natural organic compounds, are novel potential compounds for the treatment of pancreatic cancer with CTL1 inhibition. 

## Supplementary Materials

The following are available online at https://www.mdpi.com/1422-0067/21/15/5190/s1, Figure S1: CTL1 (SLC44A1) and CTL2 (SLC44A2) mRNA levels in normal tissue (blue) and PAAD cancer (red) of all available TCGA samples (analysis by UALCAN website). Figure S2: Real-time PCR analysis of the mRNA expression of choline acetyltransferase (ChAT) in MIA PaCa-2 cells and human cholinergic neuroblastoma cell line LA-N-2.

## References

[1] Rahib L., Smith B.D., Aizenberg R., Rosenzweig A.B., Fleshman J.M., Matrisian L.M. (2014). Projecting Cancer Incidence and Deaths to 2030: The Unexpected Burden of Thyroid, Liver, and Pancreas Cancers in the United States. Cancer Res..

[2] Kleeff J., Korc M., Apte M., La Vecchia C., Johnson C.D., Biankin A.V., Neale R.E., Tempero M., Tuveson D.A., Hruban R.H. (2016). Pancreatic cancer. Nat. Rev. Dis. Prim..

[3] Siegel R.L., Miller K.D., Jemal A. (2020). Cancer Statistics, 2020. CA Cancer J. Clin..

[4] Okano K., Suzuki Y. (2014). Strategies for early detection of resectable pancreatic cancer. World J. Gastroenterol..

[5] Farma J.M., Santillan A.A., Melis M., Walters J., Belinc D., Chen D.T., Eikman E.A., Malafa M. (2008). PET/CT fusion scan enhances CT staging in patients with pancreatic neoplasms. Ann. Surg. Oncol..

[6] Mohammed S., Van Buren G., Fisher W.E. (2014). Pancreatic cancer: Advances in treatment. World J. Gastroenterol..

[7] Inazu M. (2014). Choline transporter-like proteins CTLs/SLC44 family as a novel molecular target for cancer therapy. Biopharm. Drug Dispos..

[8] Nishiyama R., Nagashima F., Iwao B., Kawai Y., Inoue K., Midori A., Yamanaka T., Uchino H., Inazu M. (2016). Identification and functional analysis of choline transporter in tongue cancer: A novel molecular target for tongue cancer therapy. J. Pharmacol. Sci..

[9] Nagashima F., Nishiyama R., Iwao B., Kawai Y., Ishii C., Yamanaka T., Uchino H., Inazu M. (2018). Molecular and Functional Characterization of Choline Transporter-Like Proteins in Esophageal Cancer Cells and Potential Therapeutic Targets. Biomol. Ther..

[10] Saiki I., Yara M., Yamanaka T., Uchino H., Inazu M. (2020). Functional Expression of Choline Transporter-Like Protein 1 in LNCaP Prostate Cancer Cells: A Novel Molecular Target. Biomol. Ther..

[11] Taguchi C., Inazu M., Saiki I., Yara M., Hara N., Yamanaka T., Uchino H. (2014). Functional analysis of [methyl-(3)H]choline uptake in glioblastoma cells: Influence of anti-cancer and central nervous system drugs. Biochem. Pharmacol..

[12] Inazu M., Yamada T., Kubota N., Yamanaka T. (2013). Functional expression of choline transporter-like protein 1 (CTL1) in small cell lung carcinoma cells: A target molecule for lung cancer therapy. Pharmacol. Res..

[13] Brogsitter C., Zöphel K., Kotzerke J. (2013). 18F-Choline, 11C-choline and 11C-acetate PET/CT: Comparative analysis for imaging prostate cancer patients. Eur. J. Nucl. Med. Mol. Imaging.

[14] Hara T., Kondo T., Hara T., Kosaka N. (2003). Use of 18F-choline and 11C-choline as contrast agents in positron emission tTomography imaging-guided stereotactic biopsy sampling of gliomas. J. Neurosurg..

[15] Urbano N., Scimeca M., Crocco A., Mauriello A., Bonanno E., Schillaci O. (2019). 18F-Choline PET/CT identifies high-grade prostate cancer lesions expressing bone biomarkers. J. Clin. Med..

[16] Glunde K., Ackerstaff E., Mori N., Jacobs M.A., Bhujwalla Z.M. (2006). Choline phospholipid metabolism in cancer: Consequences for molecular pharmaceutical interventions. Mol. Pharm..

[17] Glunde K., Bhujwalla Z.M., Ronen S.M. (2011). Choline metabolism in malignant transformation. Nat. Rev. Cancer.

[18] Newman D.J., Cragg G.M. (2016). Natural products as sources of new drugs from 1981 to 2014. J. Nat. Prod..

[19] Inazu M., Hirai K., Watanabe S., Nishijima N., Shibata K., Hase A., Gido R., Yamanaka T. (2020). Development of new therapeutic drugs for pancreatic cancer targeting choline transporter-like protein 1 (CTL1/SLC44A1). Ann. Oncol..

[20] Watanabe S., Nishijima N., Hirai K., Shibata K., Hase A., Yamanaka T., Inazu M. (2020). Anticancer Activity of Amb4269951, a Choline Transporter-Like Protein 1 Inhibitor, in Human Glioma Cells. Pharmaceuticals.

[21] Kolesnick R.N., Kronke M. (1998). Regulation of ceramide production and apoptosis. Annu. Rev. Physiol..

[22] Nganga R., Oleinik N., Ogretmen B. (2018). Mechanisms of ceramide-dependent cancer cell death. Adv. Cancer Res..

[23] Katz-Brull R., Degani H. (1996). Kinetics of choline transport and phosphorylation in human breast cancer cells; NMR application of the zero trans method. Anticancer Res..

[24] Katz-Brull R., Seger D., Rivenson-Segal D., Rushkin E., Degani H. (2002). Metabolic markers of breast cancer: Enhanced choline metabolism and reduced choline-ether-phospholipid synthesis. Cancer Res..

[25] Cai H., Erhardt P., Troppmair J., Diaz-Meco M.T., Sithanandam G., Rapp U.R., Moscat J., Cooper G.M. (1993). Hydrolysis of phosphatidylcholine couples ras to activation of raf protein kinase during mitogenic signal transduction. Mol. Cell Biol..

[26] Tesiram Y.A., Lerner M., Stewart C., Njoku C., Brackett D.J. (2012). Utility of nuclear magnetic resonance spectroscopy for pancreatic cancer studies. Pancreas.

[27] Penet M.-F., Shah T., Bharti S., Krishnamachary B., Artemov D., Mironchik Y., Wildes F., Maitra A., Bhujwalla Z.M. (2015). Metabolic Imaging of Pancreatic Ductal Adenocarcinoma Detects Altered Choline Metabolism. Clin. Cancer Res..

[28] Corbin K.D., Zeisel S.H. (2012). Choline metabolism provides novel insights into nonalcoholic fatty liver disease and its progression. Curr. Opin. Gastroenterol..

[29] Yen C.L., Mar M.H., Zeisel S.H. (1999). Choline deficiency-induced apoptosis in PC12 cells is associated with diminished membrane phosphatidylcholine and sphingomyelin, accumulation of ceramide and diacylglycerol, and activation of a caspase. FASEB J..

[30] Kato Y., Ozawa S., Miyamoto C., Maehata Y., Suzuki A., Maeda T. (2013). Acidic extracellular microenvironment and cancer. Cancer Cell Int..

[31] White K.A., Grillo-Hill B.K., Barber D.L. (2017). Cancer cell behaviors mediated by dysregulated pH dynamics at a glance. J. Cell Sci..

[32] Persi E., Duran-Frigola M., Damaghi M., Roush W.R., Aloy P., Cleveland J.L., Gillies R.J., Ruppin E. (2018). Systems analysis of intracellular pH vulnerabilities for cancer therapy. Nat. Commun..

[33] Yamada T., Inazu M., Tajima H., Matsumiya T. (2011). Functional expression of choline transporter-like protein 1 (CTL1) in human neuroblastoma cells and its link to acetylcholine synthesis. Neurochem. Int..

[34] Song P., Spindel E.R. (2008). Basic and Clinical Aspects of Non-neuronal Acetylcholine: Expression of Non-neuronal Acetylcholine in Lung Cancer Provides a New Target for Cancer Therapy. J. Pharmacol. Sci..

[35] Schaal C., Chellappan S.P. (2014). Nicotine-Mediated Cell Proliferation and Tumor Progression in Smoking-Related Cancers. Mol. Cancer Res..

[36] Ogretmen B. (2018). Sphingolipid metabolism in cancer signalling and therapy. Nat. Rev. Cancer.

[37] Morad S.A., Cabot M.C. (2018). Ceramide-orchestrated signalling in cancer cells. Nat. Rev. Cancer.

[38] Galadari S., Rahman A., Pallichankandy S., Thayyullathil F. (2015). Tumor suppressive functions of ceramide: Evidence and mechanisms. Apoptosis.

[39] Wang L.Q., Zhang P.Y., Ji L.N., Chao H. (2015). A ruthenium(II) Complex Inhibits Tumor Growth in Vivo With Fewer Side-Effects Compared With Cisplatin. J. Inorg. Biochem..

[40] Johnson T.W., Gallego R.A., Edwards M.P. (2018). Lipophilic Efficiency as an Important Metric in Drug Design. J. Med. Chem..

[41] Garcia P.L., Miller A.L., Yoon K.J. (2020). Patient-derived xenograft models of pancreatic cancer: Overview and comparison with other types of models. Cancers.

[42] Yara M., Iwao B., Hara N., Yamanaka T., Uchino H., Inazu M. (2015). Molecular and Functional Characterization of Choline Transporter in the Human Trophoblastic Cell Line JEG-3 Cells. Placenta.

[43] Chandrashekar D.S., Bashel B., Balasubramanya S.A.H., Creighton C.J., Rodriguez I.P., Chakravarthi B.V.S.K., Varambally S. (2017). UALCAN: A Portal for Facilitating Tumor Subgroup Gene Expression and Survival Analyses. Neoplasia.

