# Supplementary material for: Molecular and Functional Analysis of Choline Transporters and Antitumor Effects of Choline Transporter-Like Protein 1 Inhibitors in Human Pancreatic Cancer Cells
Source: Int J Mol Sci. 2020 Jul 22;21(15):5190. doi: 10.3390/ijms21155190 (PMC7432747; doi:10.3390/ijms21155190)
Supplement: Supplementary file 1 [file ijms-21-05190-s001.pdf]

Supplementary Materials

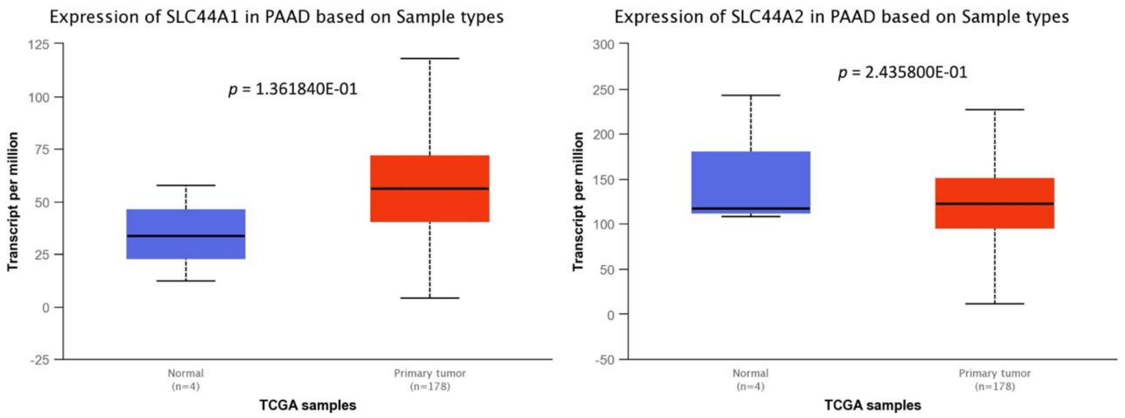

Figure S1. CTL1 (SLC44A1) and CTL2 (SLC44A2) mRNA levels in the normal tissue (blue) and the PAAD cancer (red) of all available TCGA samples (analysis by UALCAN website). Please note that low number(<10) of normal samples considered.

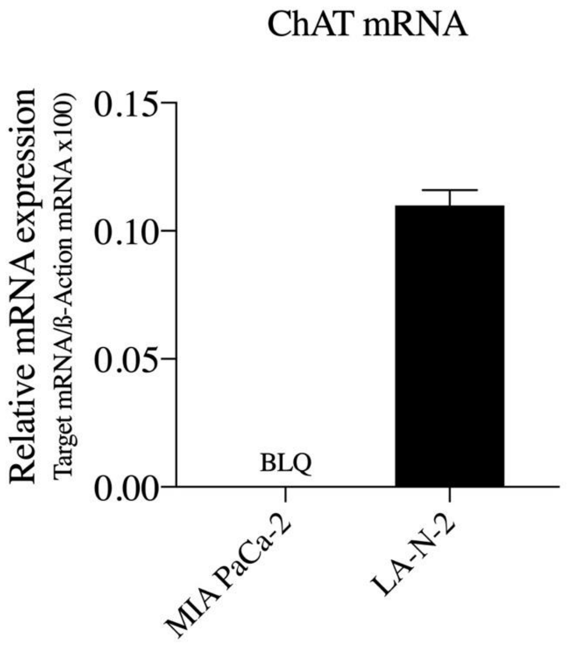

Figure S2. Real-time PCR analysis of the mRNA expression of choline acetyltransferase (ChAT) in MIA PaCa-2 cells and human cholinergic neuroblastoma cell line LA-N-2 (positive control). LA-N-2 is useful for studies on acetylcholine synthesis and secretion, and contains large amounts of acetylcholine. BLQ means below the limit of quantification.
